# Advances in delivery systems for CRISPR/Cas-mediated cancer treatment: a focus on viral vectors and extracellular vesicles

**DOI:** 10.3389/fimmu.2024.1444437

**Published:** 2024-08-30

**Authors:** Zhidu Song, Ying Tao, Yue Liu, Jian Li

**Affiliations:** ^1^ Department of Ophthalmology, The Second Hospital of Jilin University, Changchun, China; ^2^ Department of Anesthesiology, China-Japan Union Hospital of Jilin University, Changchun, China; ^3^ Department of Emergency and Critical Care, The Second Hospital of Jilin University, Changchun, China

**Keywords:** CRiSPR/Cas, cancer treatment, extracellular vesicle, exosomes, viral vectors

## Abstract

The delivery of CRISPR/Cas systems holds immense potential for revolutionizing cancer treatment, with recent advancements focusing on extracellular vesicles (EVs) and viral vectors. EVs, particularly exosomes, offer promising opportunities for targeted therapy due to their natural cargo transport capabilities. Engineered EVs have shown efficacy in delivering CRISPR/Cas components to tumor cells, resulting in inhibited cancer cell proliferation and enhanced chemotherapy sensitivity. However, challenges such as off-target effects and immune responses remain significant hurdles. Viral vectors, including adeno-associated viruses (AAVs) and adenoviral vectors (AdVs), represent robust delivery platforms for CRISPR/Cas systems. AAVs, known for their safety profile, have already been employed in clinical trials for gene therapy, demonstrating their potential in cancer treatment. AdVs, capable of infecting both dividing and non-dividing cells, offer versatility in CRISPR/Cas delivery for disease modeling and drug discovery. Despite their efficacy, viral vectors present several challenges, including immune responses and off-target effects. Future directions entail refining delivery systems to enhance specificity and minimize adverse effects, heralding personalized and effective CRISPR/Cas-mediated cancer therapies. This article underscores the importance of optimized delivery mechanisms in realizing the full therapeutic potential of CRISPR/Cas technology in oncology. As the field progresses, addressing these challenges will be pivotal for translating CRISPR/Cas-mediated cancer treatments from bench to bedside.

## 1 Introduction

Cancer is a challenging disease with high mortality rates and significant global concern. Malignant tumors pose a threat to the lives of thousands of human beings, as they are responsible for one out of every six fatalities worldwide (1). Although there have been numerous noteworthy advancements in the field of cancer therapy, such as chemotherapy, surgery, targeted biotherapy, radiotherapy, and new combination therapies, quality of life and survival time are still hindered by high post-operative recurrence rates, harmful toxic side effects, and radiation/chemotherapy resistance (2). Significant progress has been made in the treatment of malignant tumors with the replacement of traditional chemotherapeutic agents with molecular targeted medicines, which offer excellent specificity and efficacy. However, clinical application is often limited by dramatic but short-lived tumor regressions and high costs, which restrict the overall benefits (3). Therefore, a thorough comprehension of cancer biology is required for the development of novel anti-cancer treatments with fewer adverse effects. The most recent developments in sequencing technology have enabled more effective and cost-effective study of the cancer genome than ever before. A comprehensive understanding of an individual’s genome can be achieved through the implementation of an integrated strategy that integrates genomic and transcriptomic advancements. Studies have shown that cancer is a potentially fatal disease characterized by the accumulation of multiple genetic mutations and widespread epigenetic alterations throughout the genome (4). Gene mutations in cancer typically drive disease progression and influence the future course of tumorigenesis (5). Over the past two decades, high-throughput sequencing technology has identified numerous genes associated with cancer initiation and progression (6, 7). Based on advancements, gene editing technology holds great promise for cancer treatment by enabling the modulation of gene expression and correction of mutations, potentially leading to significant breakthroughs in precision oncology.

Various genomic engineering tools, such as zinc finger nucleases (ZFNs) and transcription activator-like effector nucleases (TALENs), have been used in cancer therapy to target specific DNA domains. In the 1990s, ZFNs were used for site-specific gene editing. ZFNs comprise a DNA-binding domain and a DNA-cleavage domain. The DNA-binding domain includes an array of Cys_2_His_2_ zinc fingers (ZFs), with each ZF unit containing approximately 30 amino acids that bind a single zinc atom and recognize 3bp of DNA (8). The DNA cleavage domain is derived from the *FokI* restriction endonuclease, which functions as a dimer to target specific sites and enable effective genome editing (9). The gene targeting efficacy of the ZFN technology is significantly high, ranging from 10% to 30%. ZFN is the most well-established first-generation gene editing technique. It is evident that the utilization of a ZFN-mediated approach is facilitated by oncogenes and mutant tumor suppressor genes. Indeed, p53 mutation replacement and downregulation of particular growth factors have both been accomplished using ZFNs. In K562 cells treated with and without vinblastine, an OPEN-driven ZFN strategy induced gene alteration with 7.7% and 54% efficiency, respectively, in targeting the tumor angiogenic factor VEGF-A (10). Furthermore, HEK293T cells and the SF268 human cancer cell line were used to test the effectiveness of a yeast-one-hybrid four-finger ZFN intended to replace mutant p53 with wild-type p53 (11). Nevertheless, the recognition domain of ZFNs is influenced by context. Interactions between its amino acid repeats can diminish the specificity and efficiency of gene targeting (12). In practice, designing a suitable ZFN for any specific target gene is challenging, meaning not all genes in the genome can be edited using ZFNs. Moreover, ZFN technology can cause off-target effects, which may lead to cytotoxicity (13). Due to the challenges of commercial synthesis and usability, ZFNs have gradually been replaced by other editing systems.

TALENs are another type of engineered nuclease that offer better specificity and efficiency compared to ZFNs. Like ZFNs, TALENs consist of DNA-binding and DNA-cleavage domains (14). The *FokI* endonuclease is the source of the DNA-cleavage domain of TALENs. This enzyme is capable of cleavage, but it operates exclusively as a dimer to cut the target DNA. The distinction is that TALEN fuses with the *Fok I* restriction endonuclease using a transcription activator-like effector (TALE) rather than a ZF. TALE proteins, originally discovered in Xanthomonas bacteria, typically consist of a tandem array of 15 to 19 modules. Each module, containing 34 amino acid residues, recognizes specific 1-4 bp nucleotide sequences. The enzyme is capable of targeting specific DNA sequences with a relatively high degree of precision by altering the arrangement of the modules (15). The TALEN approach showed an even greater targeting efficacy (20%–60%) than the ZFN technique. Additionally, designing a pair of TALENs for a specific DNA sequence is generally easier than designing ZFNs. This approach also provides greater specificity, with minimal off-target effects and lower cytotoxicity. Recent research has also demonstrated that TALEN gene editing technology, which is used to remove genes from cancer cells [including cells from prostate cancer (16), breast cancer (17), and hepatocellular carcinoma (HCC) (18)] provides an effective and versatile platform for investigating gene mutations at the molecular level. However, a major challenge with TALEN technology is the efficient cloning of large modules in series and the precise assembly of these modules in the designed order using ligase. Furthermore, the technique faces limitations in screening efficiency for identifying successfully targeted cells (19). However, assembling TALE molecular modules is complex and requires extensive sequencing, which increases costs. Moreover, TALE proteins have a higher molecular weight than ZFP proteins, making them more challenging to handle at the molecular level, despite their capability to target longer gene sequences (20).

**Figure 1:**
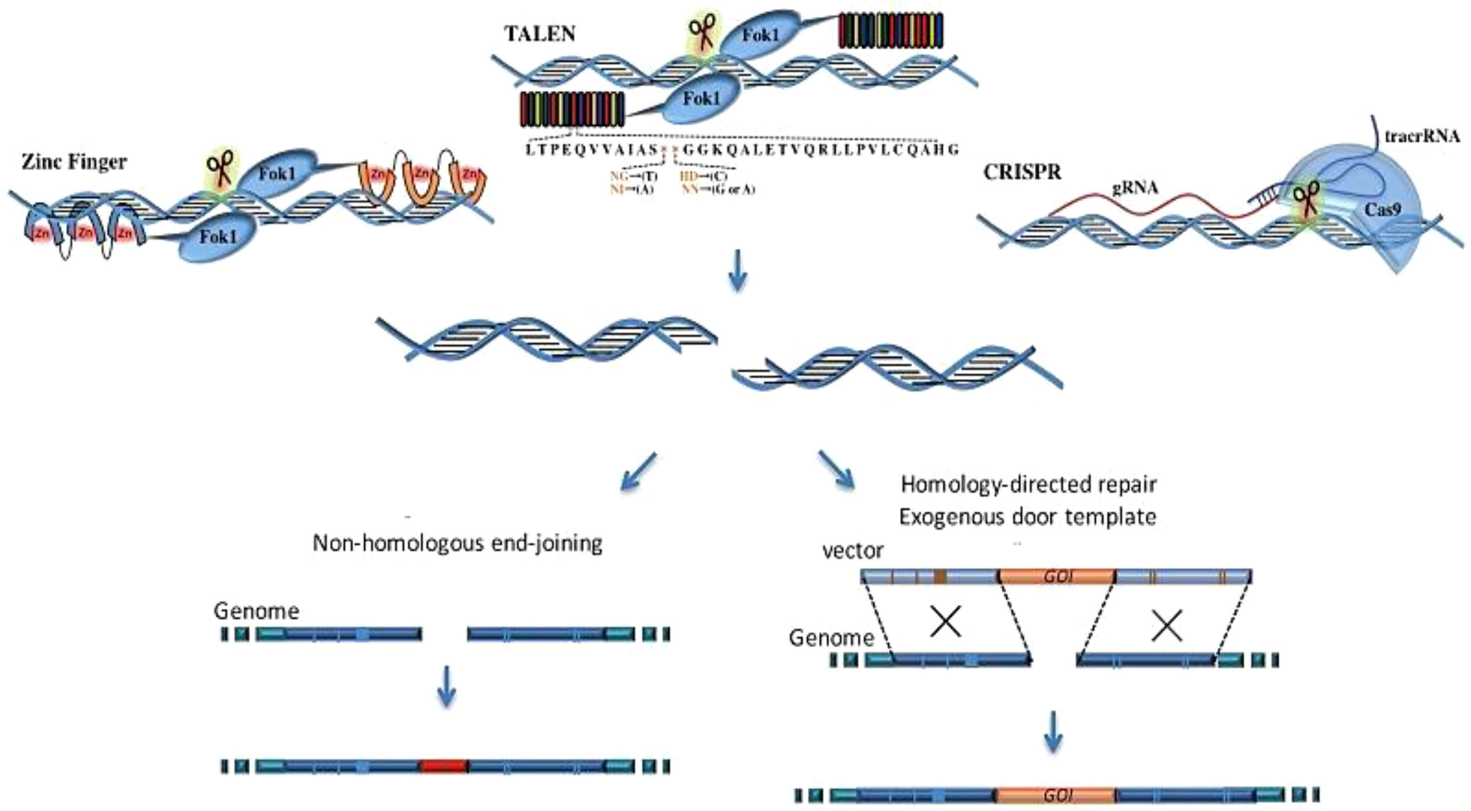
The comparison of the working mechanisms of TALEN, ZFN, and CRISPR. ZFN is composed of a zinc finger DNA-binding domain and a DNA cleavage domain of the FokI type IIS restriction endonuclease. TALEN is a similar construct that combines the FokI endonuclease with the DNA-binding domain. In contrast, the CRISPR/Cas9 system uses sgRNA to recognize site-specific DNA sequences, offering higher specificity compared to ZFN and TALEN. All these systems induce double-strand breaks (DSBs) near the targeted DNA locus, initiating DNA repair processes (28).

The CRISPR (clustered regularly interspaced short palindromic repeat) system has rapidly emerged as the leading gene-editing technology for precise modification of any selected DNA sequence. CRISPR is garnering significant attention, partly due to its potential to revolutionize medical genetics and cancer treatment. CRISPR/Cas technology offers several advantages over other nuclease-based genome-editing methods (21, 22). While other genome-editing technologies rely on protein–DNA interactions, CRISPR/Cas technology uses Watson–Crick base pairing to recognize target sequences. The CRISPR/Cas system offers several advantages, including high specificity, efficiency, the ability to target multiple genes simultaneously, and cost-effectiveness (23, 24). The transition between gene targets is significantly more efficient with CRISPR-based systems, which necessitate only the modification of the 20-nucleotide target sequence of the sgRNA for specific targeting of new genome sites, in contrast to earlier TALEN- and ZFN-based editors (25). CRISPR/Cas technology eliminates the need for protein engineering to develop site-specific nucleases for targeting specific DNA sequences, requiring only the synthesis of a new RNA molecule. This significantly simplifies and accelerates the gene editing design and implementation process (26). The CRISPR/Cas system has brought significant advancements to the field of therapeutics, particularly in cancer therapy, resulting in significant improvements (27). The working mechanism of each technique is presented in **Figure 1**.

Several strategies have been proposed based on the CRISPR technology that could be applied in cancer therapy (29). The first strategy mentioned involves gene knock-out, which focuses on eliminating the activity of a gene responsible for stimulating tumor growth. For instance, CRISPR-induced MYC gene knockout is considered a potential option to help tumor suppression. Abnormal MYC gene expression is commonly observed in a variety of cancers and disabling of this gene possibly could inhibit or even stop the spread of cancer (30). Another approach relies on enhancing one’s own natural immune response to cancerous cells. Scientists, for example, have utilized CRISPR-based gene editing to make engineered T cells with an absence of or reduction in the expression of PD-1 which boosts their killing function against cancer cells (31). In addition, CRISPR gene modification has the potential to correct the genetic mutations that are responsible for cancer, such as the inherited types that may emerge as a result in BRCA1 and BRCA2 (32). Specifically, a technique known as CRISPR/Cas9 could be used to correct BRCA1 mutations in human cells and this could serve as an initial basis for cancer therapy (33). In a research model of lymphoma, the lack of the MYC oncogene dilutes the growth of tumors. Moreover, the PD-1 gene in T-lymphocytes is upregulated which ultimately helps in targeting and killing cancer cells. Research at the pre-clinical level is quite encouraging (34), but many challenges will need to be addressed first for CRISPR-based cancer therapy to emerge as a viable alternative (30).

The delivery mechanism provides a crucial function in the therapy of CRISPR/Cas9. Delivering the CRISPR/Cas system to target cells, both *in vivo* and *in vitro*, presents a significant challenge that must be addressed before the technology can be translated into clinical applications (35). Several breakthrough delivery systems have been explored, including viral vectors, exosomes, and functional nanocomposites (36, 37). Viral vectors and EVs have been shown to be compatible with human cells and possess advantages of safety, masking of risks, capacity, and targeting (35). However, nanocomposites feature conformability and interplay facilities with multiple functional substances, therefore they are suitable for targeted delivery purposes. The type of delivery method selection is vital for the sake of achieving precision as well as the effectiveness of CRISPR/Cas9 delivery. There are, however, several unresolved issues, including off-target effects, DNA repair mechanisms, and the safe and reliable delivery of the treatment to the correct location. CRISPR/Cas-based therapy is one of the most effective methods currently available but it has its own drawbacks and complications regarding its delivery mechanisms.

This paper covers the progress of CRISPR/Cas delivery for the diagnosis and treatment of cancer, with a special emphasis on EVs and viral vectors. We ponder the problems and prospects of each of the platforms under consideration, concentrating on their projected usefulness in clinical work and personalized cancer prevention. Also, we delve into the most recent preclinical and clinical investigations with CRISPR-Cas-based approaches to the eradication of different cancer types, underlining CRISPR’s ability to transform what is yet to come with the treatment of cancers.

## 2 CRISPR/Cas-mediated cancer therapy: mechanisms and application

CRISPR-based technology holds the promise of transforming the approach to cancer treatment, enabling meticulous and effective alteration of the genome to pinpoint particular genetic mutations responsible for the proliferation and dissemination of tumors (38). It also offers a promising approach for employing gene therapy and immunotherapy in the treatment of cancer. CRISPR/Cas systems utilized in cancer treatment methodologies predominantly rely on Cas nucleases (Cas9, Cas12a, and Cas13a) and their orthologs (39). Identifying target genes is the first step in the sequential process of CRISPR screening. Next, it delineates the process of constructing and formulating CRISPR-guided RNA libraries, which are crucial for the precise targeting of genomic regions. The final step involves introducing CRISPR elements into target cells, showcasing the techniques employed for gene editing in different cell types. The subsequent stage depicts the application of selective pressures to identify the cells with desired genetic modifications, ultimately leading to the evaluation of the screening outcomes.

### 2.1 Mechanism of genome editing by CRISPR/Cas9

Barrangou et al. (40) performed *Streptococcus thermophilus* infection trials and proved that the CRISPR/Cas9 system protects bacteriophages, hence, providing an experimental confirmation of its immune function. In general, the adaptive immune response mediated by CRISPR/Cas progresses through three main phases: acquisition, transcription, and overshadowing (41). The inference of foreign DNA by the host CRISPR locus is a key condition for both crRNA maturation and Cas protein expression; the latter is responsible for the cutting of the desired sequences based on RNA guidance. In this system, the Cas9 nuclease works together with gRNA, which is a composite of CrRNA and tracrRNA, to provide the complementary pairing with DNA target sequences, resulting in site-specific double-strand breaks (DSBs) in the DNA. This procedure was simplified by the introduction of the unified RNA complex (sgRNA) which contains crRNA and TracrRNA together. This tandemly-encoded sgRNA-Cas9 framework is simplified such that a single plasmid can produce multiple sgRNAs which can target up to several loci. The 5′ end of the sgRNA binds complementary bases that designate DNA target sequences *via* Watson–Crick base pairing, whereas Cas9 affixes to the 3′ end of the sgRNA, inducing the formation of a double-strand break (DSB) at the target location 3 bases upstream of the protospacer adjacent motifs (PAMs). Structural analysis of *Streptococcus pyogenes* Cas9 (42) revealed that a specific conformational change in the RNA-DNA binding could facilitate a double-strand break mechanism.

**Figure 2:**
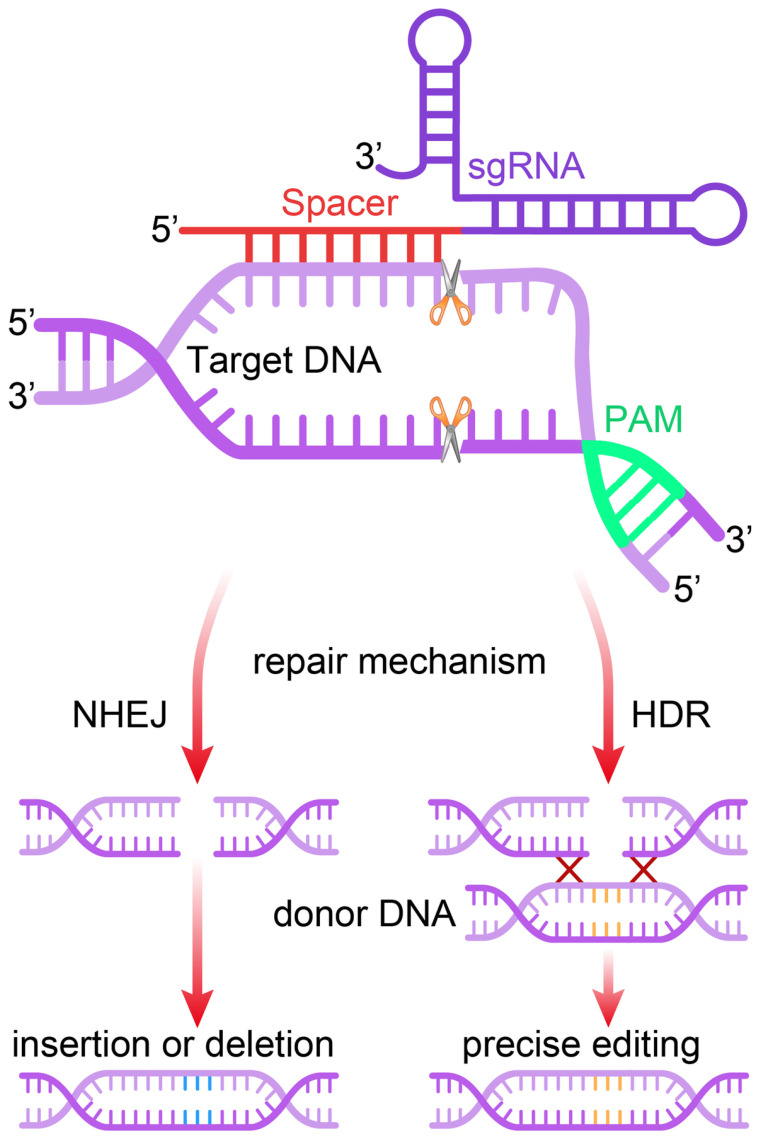
Mechanism of the CRISPR/Cas9 gene editing system. The single guide RNA (sgRNA) directs the Cas9 nuclease to a complementary genomic sequence, where Cas9 induces a double-strand break (DSB). The target sequence must be adjacent to a 5′-NGG-3′ protospacer adjacent motif (PAM) for Cas9 activity. The DSB is repaired either by non-homologous end joining (NHEJ) or homology-directed repair (HDR), the latter of which can utilize a DNA repair template to introduce precise genetic modifications or exogenous sequences (46).

Site-specific cleavage occurs when the targeted protospacer sequence matches the crRNA (or sgRNA) pairs. This cleavage happens when short motifs or protospacer-adjacent motifs (PAMs), which align with complementary parts of the DNA, are present in the target DNA (43). Without PAM, the Cas9 complex cannot distinguish between fully complementary with the desired target sequence (44). Implementing the PAM within sgRNAs is critical. Research has proved that PAM is involved in adaptive and interfering mechanisms in type II systems. When only DNA-sgRNA hybrid formation and PAM binding occur, the RuvC and HNH nuclease domains of Cas9 become especially important and responsible for the induction of DSBs in the target sequence (45). In summary, the prerequisites for identifying target DNA include the following: 1) The unique similarity consists of 20- nucleotide sgRNA sequence (crRNA) and the complementary of DNA target binding; 2) NGG PAM nearby the target sequence is considered essential (42). The DNA repair system of the cell comes into play in the final stage and tries to fix the damage. NHEJ and MMEJ are responsible for the insertions and deletions of genes (INDELs). Subsequently, this reduces the length of protein-coding regions, which is particularly fatal. The repair template comprises of the target gene and their homologs, respectively. Homology-directed repair (HDR), by way of donor DNA template, can integrate genes using these sites as cleavage points (**Figure 2**). Similarly, cases involving RNA follow analogous principles. Several RNA-targeted Cas9 systems have been developed, enhancing the functionality of the Cas9 system (47).

### 2.2 Inactivation of cancer-causing genes

The strategy of CRISPR germline mutation to eliminate oncogenes begins with selecting the exact oncogenes that are critical for cancer formation. Many of these genes occur in tumors that contain various genetic mutations or abnormal gene amplifications which eventually lead to the overproduction of their corresponding proteins. This, in turn, results in uncontrolled cell proliferation and cell growth (48). Once the target oncogene is identified, researchers design a gRNA that specifically binds to the amplified region of the target gene to deactivate it (49). One approach is gene silencing, which targets tumor-promoting genes. However, the NYC oncogene MYC has been proven to inhibit tumor growth in lymphoma animal models (50). Similarly, the CRISPR/Cas system has been employed with the purpose of depleting the oncogene E6, reactivating tumor-suppressive protein p53, and inducing apoptosis in cervical cancer cells (51). This strategy is based on the principle that cancer cells contain genetic mutations causing overexpression of oncogenes, which in turn stimulates cell growth and proliferation. This, in turn, wipes out the production of these oncogenes that lead cancer cells to persist (52). CRISPR-based approaches, as versatile tools, can be seamlessly integrated with other cancer treatments to further optimize outcomes and enhance treatment effectiveness (53). Hence, CRISPR acts together with chemotherapy, leading to the precise editing of genes which are responsible for drug resistance and thus causing cancer cells to respond better to chemotherapy (54). In addition, CRISPR technology may be used to modify patient-derived killer cells containing chimeric antigen receptors (CARs), which further improves their function as a treatment of cancer using CAR-T cells together (52). In addition to that, CRISPR allows improvement of immunotherapy efficiency through the interruption of the checkpoint genes of cancer cells by means of immune checkpoint inhibitors (55).

An additional instance comprises combining CRISPR with focused treatments, where simultaneously targeting several key pathways through gene manipulation can overcome resistance and enhance the impact of specific medications (56). Researchers can increase the effectiveness of diverse cancer treatments by modifying tumor cells or the tumor microenvironment using CRISPR, a technique that enhances drug delivery. This demonstrates how CRISPR can work together with other cancer therapies, highlighting its versatility. Moreover, these advancements pave the way for more personalized and efficient treatment strategies in the ongoing battle against cancer (57).

### 2.3 Cancer immunotherapy

Immunotherapy for cancer, an emerging category in cancer therapeutics, denotes the formation of highly directed and intensified immune response against various types of cancers (58). The core mechanisms driving cell reprogramming involve immune regulation and immune cell function, which are enhanced by genome-editing tools. Amending immune responses by using monoclonal antibodies and adaptive cell immunotherapy have shown impressive responses in the extensive stages of tumors that were considered incurable (59). Different forms of immunotherapy have been invented including active immunotherapy, passive immunotherapy, and a combination of both (60). The CRISPR/Cas9 mechanism, an extension of this pathway, is expected to lead to new research and therapeutic possibilities. CRISPR/Cas9, a component of the bacteria immune system, has undergone research to evaluate its capacity to make correct changes in the genomes. Using this approach, investigators can explore the main reasons for cancer genesis, point out the therapeutic targets, and design cell-based therapeutics (61). Given its ability to detect new targets for specialization and determine interactions between chemical and genetic, CRISPR/Cas9 is therefore capable of offering useful information on what the tumors respond to best. Moreover, it not only provides the tools to enhance immune cells and oncolytic viruses for immunotherapy but also facilitates additional advancements in the field. The precision of the technology not only in laboratory animals but also in human testing, may apply to therapeutic research (62).

### 2.4 The CRISPR/Cas9 era in cancer treatment

#### 2.4.1 Brain cancer and CRISPR/Cas9

Whether it is a young male or an old female with cancer of the brain, the highest mortality rate is always recorded (63). For the past half a century, for instance, the same medical approaches have been applied against gliomas and brain tumors (64). When fighting genetic barriers, scientists using the CRISPR/Cas9 technology do so in a fast and effective way (65). The recent study on human brain cancer, for instance medulloblastomas and gliomas, included four types of animal models, which include CDXs (66). The *in vivo* model used mice (67), and both PDXs and genetically engineered mice were used. The CRISPR/Cas genome editing approach was used, resulting in deletions of the tumor-causing Nf1, Pten, and Trp53 genes, and the Ptch1 gene that was related to the medulloblastomas. This can be achieved by targeting the genes that have been identified as playing a crucial role in the formation of brain tumors.

Out of genetic editing of the organism’s parental lineage, mutation of presumed cancer genes or tumor suppressors is one of the main approaches that scientists used to reproduce cancer, such as brain tumors in mice (68). The origin of glioblastoma is believed to be linked to mutations that result in gain-of-function in oncogenes and loss-of-function in tumor suppressor genes. The classical knockdown techniques based on homologous recombination pose a risk of cancerogenesis and gene loss of tumor suppressors (65). Nevertheless, the laborious process of generating GEMMs and the limitations due to gene duplication heterogeneity reduce its effectiveness. CRISPR/Cas9-engineered endonucleases thus manage to make the genetic double-stranded breaks at the desired targets precisely and effectively. CRISPR/Cas9 primarily offers the advantage of rapidly producing GEMM models. By contrast with other experimental genetic engineering models used before, this versatile technique offers a greater understanding of diseases than those offered by conventional genetic engineering models. Moreover, this advanced platform has been successfully utilized to develop several gene-knockout models of mice, rats, and other animals (69).

#### 2.4.2 CRISPR/Cas9 in hepatocellular carcinoma

CRISPR/Cas9 has been used in various applications to introduce point mutations in different tumor suppressor genes in hepatocellular carcinoma (HCC), a type of liver cancer (70). In one approach, through hydrodynamic tail vein injections, either p53 or Pten was targeted either by itself or combined (71). In immunodeficient mice with liver tumors, the deletion of PTEN and p53 transgenes using CRE-loxP can be observed when p53 and PTEN single-guide RNAs (sgRNAs) are used together. CRISPR/Cas9 technology has had great success in combating hepatocellular carcinoma. This approach involves the use of genetically modified strains that harbor the Cyclization Recombinase Locus X, P1 (CREloxP), technology (Gardner, 2005).

In mice that carry a Fah mutation causative for tyrosinemia type I, CRISPR/Cas9 gene editing was used to correct this in the hepatocytes. The scientists selected a non-viral method of delivery for the ssDNA, the Cas9 enzyme, the sgRNA, the wild-type G nucleotide, and the homology arms that spanned the sgRNA target area. Cell regeneration occurred most rapidly in less than 1 in 250 cells. In the case of familial tyrosinemia type I, liver-specific knockout of Fah resulted in weight loss, while returning Fah to the hepatocytes reversed this effect. Nevertheless, 0.4% of hepatocytes were delivered using hydrodynamics. This prompted scientists to develop a safe and effective method of CRISPR delivery. The novel therapy of metabolic pathway formation amendment has successfully treated of hereditary tyrosinemia type I mouse model. This strategy focuses on the second phase in tyrosine catabolism by hydroxyphenylpyruvate dioxygenase. By means of hepatocyte CRISPR/Cas9 deletion of hydroxyphenylpyruvate dioxygenase, there was a conversion of tyrosinemia type III to tyrosinemia type I. Consequently, the whole liver is rapidly replaced with hepatocytes that are modified. Hydroxyphenylpyruvate dioxygenase deficiency leads to the accumulation of harmful catabolites and an abnormal concentration of tyrosine. It is the metabolic reprogramming that does not include the sustained expression of the disease-causing gene’s wild-type proteins, which could otherwise be seen as an immune reaction and limit its long term usage (72).

#### 2.4.3 CRISPR/Cas9 in colorectal cancer

It is a cancer that affects either the rectum or colon. Recent experiments to investigate the sequencing of tumors have identified several specific genes that are only affected in this type of cancer. The effect of gene alteration is most often in support of tumor progression, the characteristics of tumors, and the development of cancer (73). The use of genetically engineered mouse models serve to demonstrate the effectiveness of orthotopic organoid transplantation’s ability to correct cellular anomalies caused by Trp53 and APC tumor suppressor genes in colon epithelial cells. In addition, immunotherapy can be a choice for treatment when addressing cancer cell transformation, gene alterations, and the growth of multiclonal cancer cells (74).

Through the use of various advanced sequencing methods that geneticists have developed, researchers have unveiled essential genes that are linked to drug resistance in human malignancies. Previously some researchers used RNAi Profiling with shRNArepoir to silence specific genes (75). The CRISPR/Cas9 library system has been integrated in clinical applications because it helps overcome the low-grade quality reduction and unwanted side effects which limit the use of the approach. This system uses genomic editing technologies to identify genes that enable cancer cells to thrive, develop drug resistance, and survive. It is also employed in laboratory models and, in some cases, as a medical treatment for living patients (76).

#### 2.4.4 CRISPR/Cas9 in breast cancer

Breast cancer (BC) is the most common type of malignancy in women all over the world and it is the leading cause of cancer-related deaths among women. Breast cancer survival rates widely vary worldwide. Developed countries have >80% overall survival rate at 5 years whereas a survival rate of 40% characterizes developing countries. Breast cancer remains one of the five leading deadly cancer conditions overall worldwide. USA is leading the world in the rate of death and prevalence of breast cancer compared to other nations. In Poland, cancer is the reason for 0,17 of all ailments and 0,14 of the fatality of cancer. In 2004, the number of global fatalities due to breast cancer reached 519,000 (77, 78).

CRISPR/Cas9 is a valid method to allow investigation on and for BC as well as other cancers. Briefly, BC is concerned with the major oncogenes, among them, PI3KCA, HER2/ErbB2, and MYC. Experts have concluded that the absence of HER2 renders BT-474 and SKBR-3 cells with HER2 less likely to be viable (79). In addition to the proteome aiding in cancer targeting, the kinome also plays a significant role. The kinome makes the family of protein kinases engage in the process of phosphorylation with proteins and fats (80). There are several universally researched and inquisitive oncogenes (HER2, PI3KCA, and FGFR) that are vulnerable to the use of CRISPR/Cas9 deletion (79).

#### 2.4.5 CRISPR/Cas9 in lung cancer

The CRISPR/Cas9 technology has manifested itself as a very effective tool in lung cancer therapy, avoiding optimal drug resistance problems, and consequently improving the success of therapeutic approaches. CRISPR/Cas9 technology has been proven to be capable of the specific gene alterations associated with treatment resistance with lung cancer caveolin-1 (CAV-1). Current data shows that CAV-1 gene disruption by using the CRISPR/Cas9 system can revert lung cancer cells’ resistance to radiation which suggests the potential of this technology to overcome the nonsmall cell lung cancer cell radio resistance and make the cells more responsive to radiation chemotherapy. In addition, a study has shown that hyperactive CRISPR-Cas9 expression increases radioresistance, underscoring the influence of CRISPR/Cas9 on gene regulation and an effect on therapeutic response in non-small cell lung cancer (81).

Furthermore, CRISPR/Cas9 can also be used to study the importance of the HER3 component of human epidermal growth factor receptors in the resistance to ALK tyrosine kinase inhibitors in ALK-positive small lung cancers. Research has recently pointed out the function of ALK+ cells and cars of resistance against lung cancer (82). CRISPR screening technologies have also been used to identify and target patients’ underlying genetic pathways, explore new therapeutic options, and address acquired treatment resistance. As a study of the molecular characteristics of lung cancer has displayed, CRISPR/Cas9 showed its capability to expedite the process (83). The research concludes that CRISPR/Cas9 is a powerful tool for the management of lung cancer by revealing targeted therapeutic procedures for this fatal disease.

Various types of CRISPR-based cancer treatment strategies are presented in **Table 1** along with their mechanism and possible advantages and disadvantages.

**Table 1:** CRISPR-based cancer treatment strategies.

Strategy	Mechanism of Action	Advantages	Disadvantages	Results	Ref
Tumor control gene inactivation	Disrupts cancer-driving genes, halting tumor growth and inducing cell death	Precision Targeting: CRISPR ensures specific gene targeting, thereby increasing treatment accuracy. High Efficacy: Promotes tumor regression and prolonged survival.	Risk of Off-Target Effects: Potential unintended genome alterations may occur. Delivery Challenges: Difficulty reaching specific tumor sites.	Demonstrated KRAS Inactivation in Lung Cancer Model with Tumor Regression.	(84)
Immune Response Enhancement	Modifies immune cells to recognize and eliminate cancer cells, enhancing natural defense mechanisms	Strengthens Natural Immunity: Amplifies the body’s defense against cancer cells. Reduced Toxicity: Offers a less toxic alternative to chemotherapy.	Potential Toxicity: Edited immune cells might damage healthy tissues. Limited Cell Availability: Obtaining sufficient cells for editing can be challenging.	Achieved Complete Remission in 2/3 Patients with Refractory Lymphomas.	(85)
Genetic Mutation Repair	Corrects cancer-related genetic mutations, potentially providing long-term therapeutic benefits	Precision Correction: CRISPR targets specific mutations, promising long-lasting effects. Potential Benefits: Correcting mutations may yield lasting advantages.	Off-Target Risks: Unintended genome changes may pose risks. Delivery Complexity: Administering therapy to precise tumor sites remains challenging.	Demonstrated Correction of BRCA1 Mutations in Ovarian Cancer Cells.	(86)
Targeted Molecule Delivery	Utilizes CRISPR to engineer viruses/bacteria for precise cancer cell targeting and therapeutic delivery	Precision Targeting: Directs therapeutic agents precisely to tumor cells. High Effectiveness: Results in tumor regression and enhanced survival.	Off-Target Concerns: Unintended genetic alterations may arise. Limited Resource Availability: Obtaining specific vectors can be challenging.	Achieved Tumor Regression *via* CRISPR-Mediated Delivery of Therapeutic Agents.	(85, 86)

## 3 Extracellular vesicles as vehicles for CRISPR CAS9 delivery

**Figure 3:**
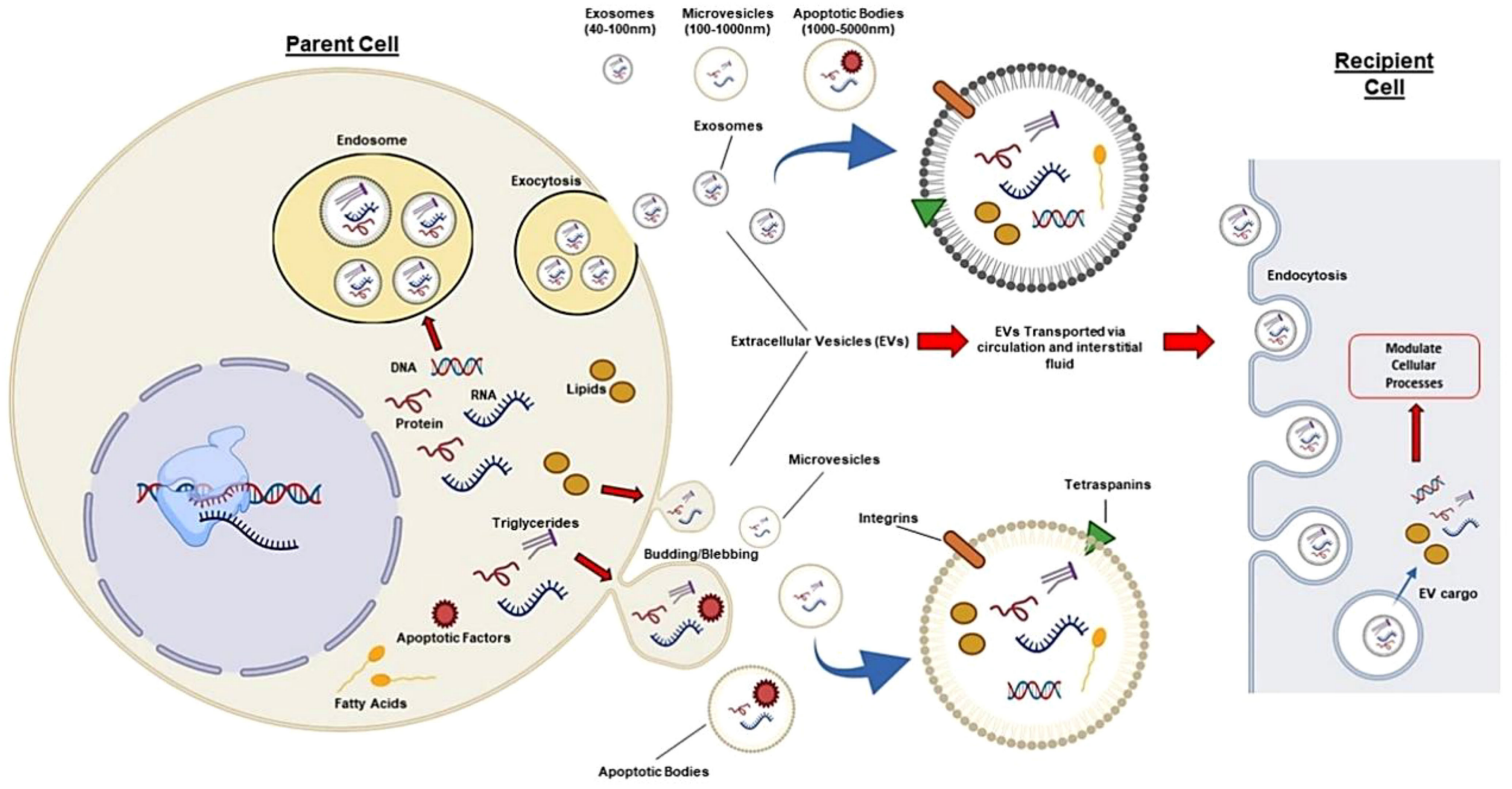
Different EVs (microvesicles, exosomes, apoptotic bodies) can be isolated from human cells and transport proteins, DNA, and RNA. In circulation, they target nearby cells and tissues. EVs have common surface markers (CD63, CD9, CD81, CD82) and specific markers such as integrins for targeting. EVs can affect recipient cells through receptor interactions or by releasing biological cargo into the cytoplasm. Thus, exosomes like the CRISPR/Cas system can be modified to target specific tissues and deliver desired cargo, enabling autologous tissue-specific gene editing (96).

It is difficult for the gRNA and Cas9 proteins to pass the cell membrane because of their respective negative charges and large molecular weights (160 kDa, respectively) (87, 88). To carry out successful gene editing in the *in vitro* CRISPR/Cas9 system, it is necessary to transport sgRNA and Cas9 protein into cells, where they can endure degradation and enter the nucleus (89). The CRISPR/Cas9 system may be neutralized or degraded in the complex *in vivo* microenvironment as a result of the immune response or other physical, chemical, or biological constraints (90, 91). Hence, to enhance the efficiency, safety, and precision of gene editing, careful consideration must be given to the delivery method and form of the CRISPR/Cas9 system. The method of direct injection of the CRISPR/Cas missiles for the *in vivo* gene editing treatment generates many obstacles. Strategies for optimizing this methodology are being worked on at present. Extracellular vesicles (EVs), nanoscale nonviral transporters, have a wide scope of different functions and are used for targeted delivery. Lipid coated nanoparticles, produced by several cells, inherently provide conveyance of cargo as well as genetic material and proteins, among cells. Given the advancements in EV research, it is clear that these extracellular vesicles play a crucial role in facilitating communication between cells (92). Furthermore, because of their participation in a multitude of physiological and pathological processes, which include immune responses, tissue repair, and cell growth, EVs have acquired a weight of consideration in the fields of diagnostic biomarkers and therapeutic applications (93). EVs can be grouped into three classes such as microvesicles, exosomes, and apoptotic bodies. Packing capacity, emerging of the genetic material, functioning, and leaving the cells, may all differ among them. Exosomes between 30 and 100 nm in size, including CRISPR/Cas9 systems, are among the more promising carriers of molecular mediators and protein-related products (94, 95). Ectosomes, or so-called microvesicles, are the expression of a plasma membrane bulging outwards. They have diameters from 100 to 1000 nm. Apoptotic bodies are the third type of EV, also referred to as apoptotic bodies which arise when apoptotic cells cleave. These bodies, with a diameter of 1000 nm and up to 5000 nm, encompass the whole size spectrum. Regardless of how they were created, they all have a similar bilayer membrane like the plasma membrane (**Figure 3**) (97).

Gene manipulated EVs are engineered to deliver CRISPR/CasRx and gRNA effectively to targeted cells for precise and site-specific gene editing. These can open up new horizons for gene therapy in curing acute diseases. Among different CRISPR/Cas systems which are EV based, including endogenous EVs, engineered EVs, and hybrid EVs, the most effective protocol could be employed for addressing targeting biases and immunogenicity issues (98). These evolutions of the CRISPR delivery system through EVs have strong potential to create precision medicine in healthcare.

Utilizing EVs to load CRISPR/Cas9 presents a promising strategy to address safety and stability concerns associated with viral vectors. The method of delivering the CRISPR/Cas9 system is critical in determining the effectiveness of gene editing. RNPs accelerate gene editing by bypassing transcription and translation processes, leading to greater efficiency and reduced off-target effects upon comparison with DNA and RNA forms of the CRISPR/Cas9 system, although they are more costly (99). Recent research has demonstrated that EVs are capable of transporting and delivering DNA, RNA, and RNP forms of the CRISPR/Cas9 system for gene editing *in vitro* and *in vivo*. For instance, EVs derived from tumor cells have been used to deliver CRISPR/Cas9 plasmids to inhibit poly (ADP-ribose) polymerase-1 (PARP-1) (100). Additionally, Lin et al. developed hybrid nanoparticles that were composed of liposomes and EVs to enhance the capacity of EVs (101). Usman et al. showed that EVs derived from RBCs were capable of transporting CRISPR/Cas9 mRNA for genome editing, and the efficacy of mRNA was superior to that of plasmids (102). Further, EVs produced from HEK293T cells have the potential to transport CRISPR/Cas9 RNPs, allowing for gene editing (103).

**Figure 4:**
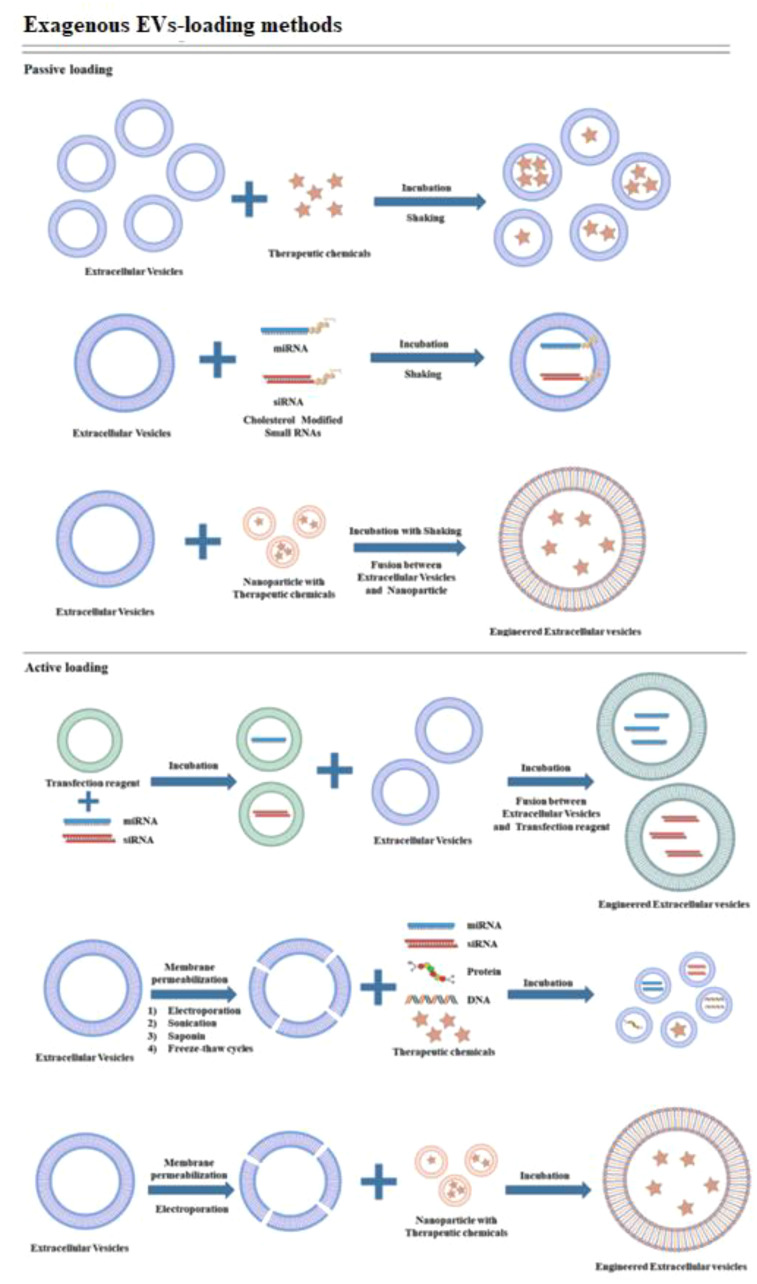
Schematic representation demonstrating exogenous cargo loading (105).

**Figure 5:**
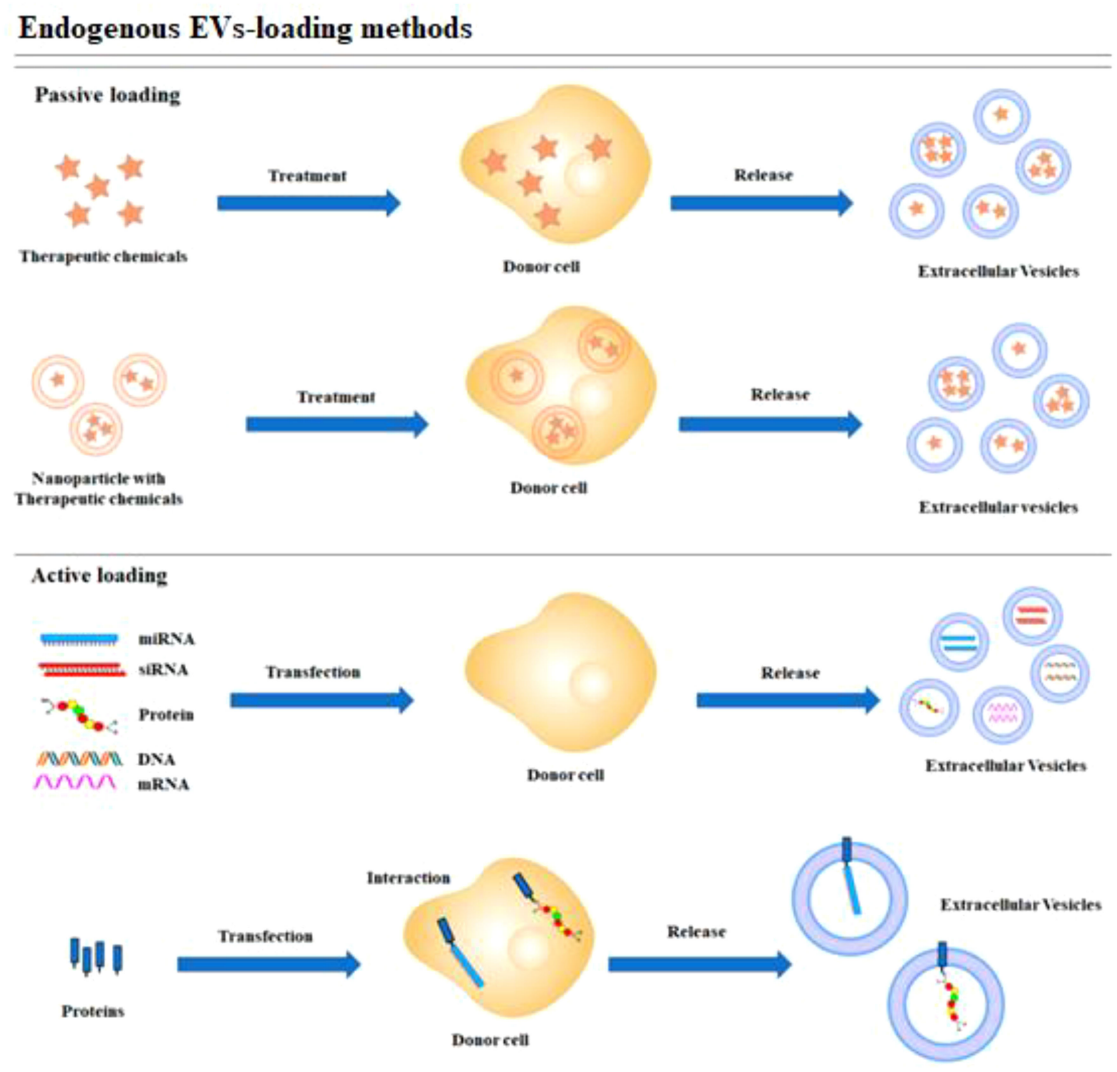
Schematic representation of endogenous loading methods (105).

In contrast, the encapsulation methods of the CRISPR/Cas9 system can be influenced by its delivery form. Effective encapsulation of CRISPR/Cas9 within EVs is crucial for successful gene editing (104). EVs should remain intact following the loading of the CRISPR/Cas9 system, in contrast to other vectors. The loading methods of CRISPR/Cas9 into EVs can be broadly categorized into two types: (1) Exogenous loading involves directly incorporating the CRISPR/Cas9 system into EVs through methods including incubation, electroporation, transfection, or sonication (see **Figure 4**). In contrast, endogenous loading involves transforming cells to produce EVs that naturally contain the CRISPR/Cas9 system (see **Figure 5**). While these loading methods are categorized as either passive or active, they can also be combined to achieve optimized loading effectiveness (106). Passive loading involves introducing therapeutic cargo into EVs via diffusion. In contrast, the process of active loading disrupts EV membranes using techniques like electroporation or sonication, facilitating cargo entry. Afterward, the membrane is restored by incubating the EVs at room temperature or 37°C, which helps stabilize them (107).

Currently, CRISPR/Cas9 plasmids and mRNA can only be incorporated into EVs using exogenous loading methods (100–102). In contrast, CRISPR/Cas9 RNPs can be loaded into EVs using both exogenous and endogenous loading methods (108–110). Loading endogenous cargoes with larger molecular weights, including CRISPR/Cas9 RNPs, remains a challenge and is still under investigation (111). Several researchers have attempted to overcome this challenge by studying strategies to improve RNP integration into EVs via specific interactions between modified RNPs and EVs. For instance, Wang et al. developed EVs with arrestin domain-containing protein 1 (ARRDC1), which interacts with WW-domain-containing proteins. As a result, Cas9 was linked to WW domains, improving its enrichment in EVs without affecting its activity or function (103). Yao et al. showed that enriching RNPs in EVs could be achieved through the interaction between RNA aptamers and aptamer-binding proteins (ABPs) (112). Additionally, myristoylated Cas9 enhanced accumulation in EVs, leading to greater RNP enrichment. The data indicate that these engineered EVs exhibit enhanced efficiency and safety as a delivery vehicle for genome editing (104). RNPs can also be enriched in EVs using light-induced protein heterodimerization techniques. Employing Cryptochrome 2 with CD9 or Myristoylation-Palmitoylation-Palmitoylation lipid modification facilitated efficient loading, achieving approximately 25 Cas9 molecules per EV. Using this method, the Cre reporter cassette was able to achieve a gene editing efficiency of 51% in HEK293 cells and 25% in HepG2 cells, demonstrating high functional delivery. The therapeutically important PCSK9 gene was also effectively targeted and knocked down by this method, resulting in a 6% indel efficiency in HEK293 cells (111). Li et al. successfully loaded C/EBPα CRISPR/Cas9 into EVs (113). Due to the fact that RNA-binding proteins can improve RNA incorporation into EVs (114), they fused the RNA-binding protein HuR with the exosomal membrane protein CD9 to improve loading efficiency. To engineer the CRISPR/Cas9 cargo, they transfected HEK 293T cells with CD9-HuR, CRISPR/Cas9, and the packaging plasmids psPAX2 and pMD2G, followed by EV isolation. Furthermore, it was found that CD9-HuR exosomes could effectively enrich functional miRNA inhibitors or CRISPR/dCas9, particularly when the RNAs were modified with AU-rich elements. In summary, a novel strategy has been established for improving RNA cargo encapsulation in engineered exosomes, enhancing their functionality in recipient cells (113). Beyond the aforementioned loading methods, the CRISPR/Cas9 system can also be introduced into EVs using anthrax lethal toxin. This toxin comprises protective antigen (PA) and lethal factor (LF). PA forms a channel that recruits and transfers LF. This system allows for the delivery of foreign proteins fused to the N-terminus of LF, such as the Cas9 protein, into cells. To demonstrate the technology’s potential, the CRISPA system was employed to silence lipolysis-stimulated lipoprotein receptor (LSR) in HCT116, a human colon cancer cell line, and green fluorescent protein (GFP) in 293T cells, which are embryonic kidney cells showing the expression of GFP. Importantly, it has been shown that the PA transporter can be modified to bind to particular receptors on the surface of host cells, potentially allowing for optimized, cell type-selective delivery of Cas9 (115). Simultaneously, LF is transported into the cytosol and EVs, where it can be loaded with a variety of substrates, including ASOs, siRNA, LFn-DTA, and Cas9 protein (116, 117).

### 3.1 Exosome

Exosomes revolutionized the field by serving as diagnostic and therapeutic indicators for cancer. These pin-sized vesicles, that are released by the mother-cells, offer cancer specific therapies since their resemblance to the real cells makes them particularly absorbable by neighbor cells. Inhibiting this function can be utilized for specific payloads to be loaded in tumor-derived exosomes, thereby allowing cell-specific cancer therapies to be designed using a targeted approach. The authors demonstrated in a previous study that if the exosomes which originate from tumor tissues are filled with doxorb, a new cancer drug, and given over the entire body, then the antitumor impact would be increased as compared to using only doxorb in treatment (118). There was an advanced method used which involved applying mesoporous silicon nanoparticles (PSiNPs) which consist of biocompatible porous silicon and were loaded with doxorubicin (DOX@E-PSiNPs) into isolated tumor cells. Then, the animals were randomly divided into different treatment groups and injected intratumorally with the prepared DOX@E-PSiNPs in exosomes. Using the model system, this group showed that exosomes were internalized by both the main tumor cells and cancer stem cells (CSCs), the outcome of which was a significant decrease in tumor growth (119). Using exosomes to deliver CRISPR/Cas systems to cancer cells shows promising prospects, as they can efficiently target and deliver the system directly to disease sites.

EVs are fundamental to a number of physiological and pathological processes by assisting immunity responses, helping to heal wounds, and providing nourishment and improvement for cells. Thus, they have increasingly addressed researchers’ needs for diagnostic biomarkers and therapeutic options. Researchers examined exosomes (vesicles) from epithelial and cancer cells. The purpose was that these vesicles conveyed an operational CRISPR/Cas9 system that accurately targets a PARP-1 (poly(ADP-ribose) polymerase-1) inhibitor. The reports gave an exact number of cells of the ovarian cancer that died through the programmed cell death as well as the exact number of cells that became sensitive to the cisplatin chemotherapy. The individually dichotomic use of the therapeutic techniques resulted in 57% reduction in cancer cell proliferation which is ~2 times as effective as the use of cisplatin and exosomes therapies separately (only 86%). A major problem in the context of gene therapies involving exosomes is that there exists a risk of side effects in the peripheral and remote tissues as well as when delivering CRISPR/Cas components.

The precise targeting of exosomes by recipient cells is the process that enhances the utility of these exosome packaging abilities. Alvarez-Erviti et al. produced artificial dendritic cells that display Lamp2b on their outer membranes and combined it with rabies viral glycoprotein (RGVP) to synthetize brain-targeting exosomes loaded with siRNA. These findings of *in vivo* delivery were confirmed in the mice whereby robust therapeutic potential was reported without non-specific uptake by other tissues (120). Another research study reflects a similar strategy of miRNA delivery to cartilage for treating osteoarthritis where it showcases significant potential for penetrating the hard-to-access tissues (121, 122).

However, exosomes differ from traditional viruses that can be modified in size. Big loads such as the CRISPR/Cas system are transported by producing hybrid exosomes *via* the mixture of natural exosomes that are synthesized from cells and artificial liposomes. Hybrid exosomes are liposome-coated, giving them a larger capability of encapsulating load. In addition, the positive charge of liposomes results in them having better interaction with RNA and DNA by means of membrane fusion. This is a means of avoiding the use of mechanical methods, such as electroporation, as a way of loading the genetic material into the exosomes (123). It should be worth noting that the changes to exosomes did not affect their ability to bind and be taken up by certain cells (124). Lin and his team developed this method to carry CRISPR/Cas9 expression vectors into mesenchymal stem cells (MSCs) (101), which are hard-to-transfect donors.

### 3.2 Key factors of EVs as carrier

With respect to the cargo transportation, EVs may have an advantage over traditional vesicles due to their significantly larger storage spaces, aside from safety considerations. Hydrophilic, gargantuan, and dynamic charges which possess naked nucleic acids and proteins leads them to these challenges (125). This consequently meant they had to be put in EVs for their transportation and protection. Moreover, a EV membrane gives both the nude nucleic acids and proteins shelter from serum endonucleases and protection from the immune system (126). EVs have a unique feature that distinguishes them from other vectors: they are capable of carrying in different types of substances; namely sugars, nucleic acids, proteins, or lipids. EVs transport a wide range of biomacromolecules, including nucleic acids and proteins, from external sources, serving various functional roles. When EVs are being formed, it is natural that an EV contains numerous biomacromolecules important to cell communication, disease progression, and for therapeutics therapies (127, 128). Different biological obstacles, such as thick tissue (for tumors and cartilage), acidic and enzymatic microenvironments (i.e., in the intestinal mucosa and stomach), and vascular endothelial cell barriers (e.g., blood–embryo, blood–brain barriers (90, 129), can limit the delivery of substances in living organisms. Due to their excellent biocompatibility and capability to be spontaneously formed, EVs can carry cargoes very efficiently, breaking through all kinds of natural biological barriers (127, 130). An excessive and complicated tumor microenvironment imposes a strong barrier to drug penetration, worsening nanomedicine’s effectiveness (129). Liposomes exhibit the ability to invade tumor tissue and transport medicines. However, internal distribution depends on various parameters including size, charge, encapsulated compound, and tumor atmosphere. Thus, the applications of liposomal encapsulation are limited (131). EVs associated with a tumor can penetrate deeply into tumor tissue, potentially accelerating its development. Sánchez and M. found that the production of metalloproteinases increased the dissolution of extra cellular seed matrices into them.

An area of EVs also examined is their surface protein overexpression. Protein levels on surface membranes of CD147, Tspan8, or CD44 and some miRNA such as miRNA-494, miRNA-542-3p, or miRNA-21-5p were found to be increased (132). In their published research, Kim et al. injected tumor-derived exosomes carrying the CRISPR/Cas9 platform to effectively penetrate tumor tissues, escaping these barriers and conducting gene editing (100). The gut mucosa has the capacity to hinder the uptake of different carriers, where a payload is transferred (90). The scientists found that the smallest size and the negative charge of vectors were the ones that showed the best outcomes in diffusion (133). These EVs, which are smaller than the others and have the same gross size, negative charge, and constant composition, are easily digested (134, 135) by the colon. Additionally, epithelial EVs easily go inside the colon which is their destination (136). By passing through the digestive system, EVs are thought to shield certain biomacromolecules from digestive enzymes such as curcumin and distinguished RNA species (90). The blood-brain barrier (BBB) has the salient function of maintaining a physiological mechanism that selectively helps the crossing of specific small molecules through the endothelial membrane excluding harmful substances or toxins (137). The BBB performs as barrier that blocks the movements of drugs and other biomacromolecules from destabilizing the brain. Nevertheless, EVs have the potential to serve as carriers for drugs and biomacromolecules, transporting them across the BBB. The qualities and indicators of the EVs decide how successful this step is (size; nature; steps; goal). Morad et al. showed that EVs of the tumor crossing the BBB in living bodies actually occurs *via* the mechanism of endocytosis. This IV experiment stimulated EVs to have Rab7 expression reduction in endothelial cells for improving their transport (138).

### 3.3 The pathological and physiological roles of EVs

EVs have a wide distribution and are responsible for transporting different biomacromolecules in an organized manner within living organisms. This transport plays a crucial role in regulating multiple physiological processes (127). Particularly in the context of immunoregulation, EVs produced by B lymphocytes can present antigens via major histocompatibility complex (MHC) proteins located on their surface (139). Recent research has specifically examined the immune-regulatory roles of EVs in tumors. Reports indicate that EVs produced from tumor cells have the capacity to regulate the immune system, either promoting the advancement of cancer or exhibiting anti-tumor activities (140, 141). Hoshino et al. have shown that during tumor metastasis, EVs originating from tumor cells fuse with resident cells in organs (such as fibroblasts and epithelial cells) to establish a conducive environment. The fusion process is made easier by the presence of integrins in exosomes, which can activate Src phosphorylated and proinflammatory S100 proteins which are IP 3. Such cellular mechanisms are responsible for further transit of cancer cells throughout the body (142). Nonetheless, numerous studies have shown that EVs derived from tumor cells are capable of migrating tumor associated antigens to the host cells, thus stimulating anti-tumor immunity by activating CD8(+) and CD4(+) T cells. The activated T lymphocytes may effectively eliminate cancer cells and hinder tumor growth progression (141).

Apart from this, EVs can modulate varied cellular communication that is implicated in the cell cycle (cell growth and apoptosis), tissue regeneration (angiogenesis and tissue remodeling), and the healing process (127). The major task of platelet-EVs is to mediate the cascade of platelet activation, simultaneously inducing cell cycle, tissue repair, and regeneration. There is a spectrum of EVs in focus containing different agglutinants, growth components, immune regulators, RNAs, and lipids (143). Kim et al. confirmed that platelet-derived EVs are capable of the formation of human umbilical vein endothelial cells (HUVECs) (144). Zhang et al. have shown in a rat model on femoral nonunion that the EVs from BM-MSCs have the capability of enhancing angiogenesis and telling the formation of bone. HUVECs that received EVs in the *in vitro* period used internalization of EVs for promotion of proliferation (145).

EVs are not limited to only being oncogenic carriers; rather they are also implicated in pathways of chronic illnesses and infectious diseases (127). EVs, successfully operating in the tumor, build a cross-cell bridge for supplying cancer, stromal, and immune cells, playing key roles in tumor formation, therapy resistance, metastasis, and immunity by delivering DNA, RNA, proteins, and metabolites. The EVs initially produced by cancer cells do this by influencing the activity of the cancer cells producing the EVs along with that of the neighboring tumor and stromal cells (140). Utilizing CML EVs, Raimondo et al. showed that these are able to induce cell proliferation and activate antiapoptotic pathways in cancer cells (146). The group of That et al. found that in glioma, a subset of cancer cells expressing EGFRvIII can generate EVs containing EGFRvIII which can later activate the AKT pathway in surrounding tumors cells in a so-called anchorage-independent fashion (147). Another work by Antonyak et al. also illustrated the fact that EVs formed by MDA-MB-231 and U87 glioma cells can transfer some cancer features in fibroblasts and epithelial fibroblasts (148).

Among recent studies, EVs are involved in different chronic or infectious disorders, such as pulmonary fibrosis (PF), cardiovascular diseases, and viral infections (127, 149, 150). PF is an intricate lung condition resulting from the abnormal proliferation of fibroblasts and an overproduction of extracellular matrix. As to the etiology of PF, there still is no clear answer; nevertheless, studies confirm a relationship between this disease and EVs (149). Yao et al. showed that the upregulation of miR-328 from the EVs of M2 macrophages promoted fibroblast proliferation in an artificial setting and the worsening of PF *in vivo* (151). EVs’ influence on atherothrombosis’ course depends on EV’s distinct categories, the types of molecules which are carried by them, and the cells that are involved in the process. Furthermore, EVs are mainly involved in all atherosclerosis stages, such as the starting of lesions, the enlargement of plaques, the rupture of plaques, and blood clot formation (150). It was revealed by RNA viruses that viral EVs transport biomacromolecules of a different kind to adjust pathological processes associated with the infection and the cellular response (127).

### 3.4 EV-mediated CRISPR/Cas9 delivery

Ran et al. tested the application of EVs to the delivery of the CRISPR/Cas9 system and potential DNA cleavage in a stable HEK293T cell line carrying the insert EGFP gene in their study. In addition, the researchers observed a remarkable decrease in the EGFP expression level, suggestive of the successful functioning of the EV-mediated DNA editing (104). A research group inserted a stop element between the promoter and DsRed, thus DsRed was used as a molecular reporting system in the lung cancer cell line A549. Through therapeutic intervention, the EV-CRISPR/Cas9 system removed the stop element (152) of the A549 cells, consequently allowing the cells to emit light. Previous works have been focused on verifying the feasibility of EV/CRISPR technology implementation in both mammalian cells and cancer cells. The research focused on specific oncogenes in EV-based cancer therapies, with the aim of improving their efficacy. Zhuang and coworkers developed a vector of EV-CRIPSR/Cas9 to target WNT 10B, a Wnt family oncogene that harbors mutations and is overactive in the HepG2 hepatocellular cancer cell line. The mouse experimentation demonstrated that injection of the vector in a dose-dependent way via intravenous route reduced the size of tumor explants (109). Acute myeloid leukemia (AML) patients frequently display miR-125b overexpression at locations causing the disease. Additional studies revealed two important processes involved in the carcinogenic process, promoting the proliferation of leukemic cells and suppressing their differentiation (153, 154). Usman and coworkers added mRNA and sgRNA to cargo-transformed EVs which were transported from red blood cells to MOLM-13 cells. MiR-125b expression decreased considerably. Reprogramed leukocytes did not differ phenotypically from cancerous cells. They have identified that EVs, which contain Mir-125b antisense oligonucleotides, suppress cell proliferation (102). The Tumor-Promoting MYC family genes play an important role in cancer outbreaks, especially Burkitt lymphomas. C-myc gene dissemination caused by chromosomal translocation in BLs (155) were identified. A study revealed that CRISPR/Cas9 endonuclease therapy with EVs also caused changes in people’s DNA that led to the MYC protein reducing apoptosis and cell proliferation. However, no *in vivo* experiments that were in the process of examining EV-delivered CRISPR/Cas9 were found (156). The G12D KRAS mutation modulates the Wnt pathway and is therefore a key component of pancreatic initiation and growth (157, 158). McAndrews along with his fellow researchers showed that KPC689 cell development was slowed down by EV cargo in a click-and-ship model. The suppression of growth of implanted pancreatic tumors in a mouse model was also demonstrated by using the EVs as a carrier of CRIPSR/Cas9 (159).

Extracellular vesicles can also be used in combination therapy with conventional cancer therapy. For instance, tumor-derived exosomes (TDEs) offer a valuable opportunity for cancer treatment due to their enhanced uptake by tumor cells, as they originate from the same tumor environment. This feature can be utilized to boost therapeutic efficacy by incorporating therapeutic agents into TDE. A recent study showed that targeting tumors with TDEs loaded with doxil led to more significant tumor regression compared to systemic administration of doxil alone (118). Exosomes possess characteristics making them ideal for delivering CRISPR/Cas genes and serve as effective delivery vehicles. For example, a clinical trial included three end-stage lung cancer patients who had developed resistance to cisplatin. The researchers found that intrathoracically injecting cisplatin-loaded EVs derived from A549 human lung cancer cells considerably decreased the number of cancer cells and the prevalence of stem cell-like cancer cells in pleural effusions. Treatment with drug-loaded EVs not only reduced the tumor burden but also extended survival more effectively than free chemotherapy. The EVs showed increased anticancer activity in drug-resistant cells, a benefit attributed to their enhanced uptake and prolonged intracellular retention, which is facilitated by the characteristic softness and deformability of resistant cells (160).

Researchers investigated the feasibility of using TDEs to deliver CRISPR/Cas9 into SKOV3 xenograft mouse models of ovarian cancer. Exosomes derived from epithelial cells were compared to TDEs in terms of their ability to deliver CRISPR/Cas9 systems and silence PARP-1 in the study. Interestingly, the combination of cisplatin and CRISPR/Cas9-loaded SKOV3-Exo resulted in a synergistic effect, reducing SKOV3 cell proliferation by 57% *in vitro*. In comparison, cisplatin alone and SKOV3-Exo alone achieved 21.6% and 30% inhibition, respectively. Additionally, the combination of cisplatin with iPARP-1/SKOV3-Exo led to a higher apoptotic rate of 27.56%, compared to 11.06% with cisplatin alone and 11.9% with iPARP-1/SKOV3-Exo alone (100). These results highlight the improved therapeutic potential of combining TDEs for CRISPR/Cas9 delivery with chemotherapy, presenting a promising strategy for enhancing the efficacy of cancer treatment. Furthermore, the efficacy of chemotherapy in targeting cancer cells can be improved by combining CRISPR with chemotherapy, which can help overcome drug resistance (161). Currently, the efficacy of CRISPR-based combination therapies in humans has not yet been completely established, and they are primarily in the preclinical stage (162). It is possible that future developments in precision gene editing techniques and delivery mechanisms might overcome some of these limitations, potentially rendering CRISPR-based combination therapies a more viable method of cancer treatment (163).

#### 3.4.1 Immunogenic and safety concern of EVs

Before employing EVs to deliver CRISPR/Cas9 components, it is crucial to assess their biosafety and potential adverse effects to ensure that EVs do not have any harmful roles (100, 101). Therefore, it is imperative to select the appropriate cell source, whether it be autologous or non-autologous, in order to ensure the safe and effective delivery of EVs. This is because EV characteristics are inherently dependent on donor cells. An autologous source ensures that the ideal materials are presented and that the same host cells are used, while also avoiding potential hazards of host immune responses and mismatched antigens (164). However, preparing EVs from autologous sources can be challenging due to issues with availability and time constraints. Establishing homologous or identical donor cell banks can provide a convenient and timely solution, enabling large-scale reserves of EVs derived from sources, such as dendritic cells (DCs) (165), serum (110), and RBC-derived EVs (102). Non-autologous sources are often preferred for their regulatory and commercial advantages, offering a streamlined, highly standardized product. These sources have proven to be safe, cost-effective, and practical for EV production (166, 167). MSCs are among the most frequently used sources for EV production. Numerous studies have revealed that MSC-derived EVs exhibit low immunogenicity *in vivo* (168). Mendt et al. demonstrated that repeated administration of MSC-derived EVs in mice did not result in significant toxicity or immune reactions *in vivo* (169). Furthermore, Zhu et al. also found that EVs derived from HEK293T cells were well-tolerated *in vivo*, with no significant immune response or signs of toxicity observed (164). However, the safety of tumor cell-derived EVs is debated, as they carry various tumor-associated biomacromolecules that could potentially influence tumor development (87). Choosing suitable MHC cell sources can help minimize or prevent unwanted immunogenicity. Additionally, it is essential to select the appropriate cargo and molecular attributes prior to EV preparation (170).

Various strategies have been suggested to reduce the immunogenicity associated with EVs. To enhance both delivery and safety, approaches combining engineered EVs with antibodies, liposomes, or extracellular polymeric nanoparticles (EPNs) have been employed (171, 172). For instance, EVs modified with chimeric proteins, such as pre-miR-199a-3p, demonstrate low immunogenicity and negligible changes in immune markers, thereby broadening the potential applications of EVs for cargo delivery (164). EV immunogenicity presents significant opportunities for developing new vaccines or vaccine vectors. By applying gene and chemical modifications, bacterial membrane EVs can be enhanced with additional functions, making them valuable for immunotherapy against both infectious and non-infectious diseases (173). Reducing the immunogenicity of CRISPR/Cas components can be achieved through strategies such as deleting genes encoding undesirable immunogenic proteins (174), removing Cas9 epitopes (175), and using appropriate Cas proteins or orthologs (176). These approaches help ensure safer and more effective targeted delivery.

#### 3.4.2 Biodistribution and pharmacokinetics of EV-delivered CRISPR/Cas9

One challenge with using EVs as a therapeutic approach for cancer is their tendency to be absorbed not only by tumors but also by normal organs and tissues, which can result in unwanted uptake by non-targeted cells. For example, after systemic administration, naturally produced EVs are often predominantly distributed to the liver, lungs, kidneys, and spleen. The primary distribution of labeled exosomes to the liver and spleen is also demonstrated by the pharmacokinetic analysis of their intravenous injection in mice, following which they are rapidly cleared from the body (177, 178). In addition, the *in vivo* drug delivery process of EVs is characterized by the continuous decomposition and release of their contents, as well as the dynamic adsorption of various molecules throughout the process (179). Therefore, for the successful development of EVs as therapeutics and drug-delivery vehicles, it is imperative to accurately predict, monitor, and control their biodistribution. The selection of an appropriate animal model is a critical factor that may affect the biodistribution pattern of EVs. Regardless of their size or origin, a significant portion of EVs is rapidly cleared from circulation in animals following administration. Typically, this clearance occurs within minutes after EV administration. This can be explained by a two-phase exponential decay model, where EVs first undergo rapid distribution to organs (distribution phase) with a short half-life (T1/2α), followed by a slower elimination phase, during which EVs are gradually cleared by the liver and kidneys, resulting in a longer half-life (T1/2β) (180, 181). Tissue-resident and monocyte-derived macrophages are key players in EV clearance. Research has shown that the rate of EV clearance from the blood is significantly reduced in mice that have been depleted of macrophages (182). The exposed phosphatidylserine (PS) on the surface of small EVs is regarded as a critical ‘eat me’ signal, facilitating their engulfment by macrophage (183, 184). Matsumoto et al. utilized annexin V, a PS-binding protein, to mask the PS present on the surface of the exosomal membrane. This approach led to a delayed clearance of the injected exosomes by macrophages (183). Employing ‘don’t eat me’ signals is another strategy to reduce clearance by the mononuclear phagocyte system (MPS). For example, CD47, which is highly expressed on erythrocyte membranes, serves as a key ‘don’t eat me’ signal. Kamerkar et al. increased the circulation time of exosomes by incorporating CD47 on their surface. This modification interfered with the interaction between exosomes and phagocytes, thereby reducing their uptake by monocytes and macrophages (185)

The targeting ability of EVs is inherently influenced by their cell of origin. The specific glycans, proteins, and lipids present in the EV membrane dictate their affinity for particular organs, tissues, and cells (128). EVs derived from lung, liver, and brain tumor cells were demonstrated by Hoshino et al. to be preferentially absorbed by lung fibroblasts, Kupffer cells, and brain endothelial cells, respectively. Additionally, this investigation demonstrated that lung tropism is associated with EV integrins α6β4 and α6β1, whereas liver tropism is associated with integrin αvβ5 (142). Furthermore, Kim et al. showed that CRISPR/Cas9-loaded EVs derived from SKOV3 cells selectively accumulated in ovarian tumors *in vivo*, in contrast to those derived from HEK293 cells. This selective accumulation is likely attributed to cell-specific tropism (100). Wan et al. found that EVs derived from hepatic stellate cells and loaded with CRISPR/Cas9 specifically targeted the liver, with no detectable presence in other organs such as the heart, spleen, lungs, or kidneys (108). Off-target distribution can be mitigated by modifying the EV membrane. For instance, Zhuang et al. demonstrated that HEK293T cell-derived EVs could be directly modified with valency-controlled tetrahedral DNA nanostructures (TDNs) conjugated with DNA aptamers. This modification enabled specific targeting of EV-based Cas9 delivery to the liver through cholesterol anchoring (109). Furthermore, a key study utilized the targeting precision of CAR (chimeric antigen receptor) to develop an EV-derived delivery platform for the CRISPR/Cas9 system. This platform was designed to specifically target the MYC gene, presenting a novel therapeutic approach for hematological malignancies (156).

Based on the above studies, it can be inferred that the natural targeting capabilities of EVs, derived from their parent cells, allow for more precise delivery of CRISPR/Cas9 components to desired cells and tissues, minimizing off-target effects. Modifications to the surface of EVs, such as adding targeting ligands or antibodies, have been explored to alter biodistribution patterns, aiming to enhance delivery to specific tissues such as the brain or tumor sites. The pharmacokinetics of EV-delivered CRISPR/Cas9 systems are influenced by the intrinsic properties of EVs, including their stability in circulation and ability to evade the immune system.

## 4 Viral vectors for CRISPR/Cas9 delivery

Viruses serve as authentic carriers for gene delivery, enabling mammalian gene therapy through recombinant and pseudotyped viral vectors. Adeno-associated viruses (AAVs), adenoviral vectors (AdVs), and lentiviral vectors (LVs) are among the most utilized viral vectors for delivering CRISPR/Cas, and they are currently undergoing clinical trials (35).

### 4.1 Adeno-associated viruses

AAVs are the smallest non-enveloped single-stranded DNA viruses which are not able to attack the human body. This viral family, Parvoviridae, have attracted the attention of researchers as the vehicle that includes the gene delivery (186). AAVs are the most common vectors used for CRISPR modifications of human genomes. Their notable safety advantages and therapeutic prospects have resulted in some AAVs being utilized in several different gene therapy clinical trials (187). AAVs also do not trigger a significant immune response in the human body. Nevertheless, their small size restricts the capacity for material packaging. They package Cas9 and sgRNA separately and deliver them both to the cells at the same time to resolve this problem. Swiech et al. injected a 1:1 mixture of AAV-SpCas9 and AAV-sgRNA (targeting MecP-2 gene) into the hippocampal dentate gyrus (DG) of adult male mice. Following viral injection, 4 weeks later, granule cells of the hippocampus exhibited the co-transduction of 80% efficiency in the two vectors and 70% gene modification efficiency of MecP-2. Other suggested researchers are about to take up the job of genetically modifying small Cas9 proteins in bacteria. According to Run et al., SaCas9 is smaller than 1 KB as compared to SpCas9 (188).

The CRISPR/Cas system activated by dCas9 (deactivated Cas9) or nCas9 (Cas9 nickase) is a base and prime editing technique. This novel approach of the CRISPR/Cas system is a monomeric vector system, which prevents the complexities associated with the lengthy and fragile viral vectors and eliminates the constraint of the restricted capacity of viral vectors for packaging (189). Moreover, this method does not induce DSB, no donor template DNA is needed, and a high editing capacity is available for non-dividing cells (190). AAVs has exhibited potentiality as a good framework for cramming CRISPR DNA with base-editing tools (186). Another investigation revealed that the double AAVs and CRISPR/Cas system’s Cytidine base editors were successfully used for the *in-vivo* treatment of ALS in an animal model. The feasibility of applying the primary AAV editor as the essential tool for counteracting harmful alleles and achieving cancer in older mice has so far been confirmed. This is so because it has a lower off-target effect compared to the base editor which is CRISPR/Cas (190, 191). Although AAV-based and prime editing techniques solve some deficiencies that arise from AAV-mediated CRISPR/Cas9 delivery, such as antibody mediated immune response, persistence of vector, and off target activity, these restrictions still remain to some degree (189).

### 4.2 Adenoviral vectors

AdVs are a group of viruses that consist of a double-stranded DNA and do not have a lipid envelope, and are further capable of infecting both dividing and non-dividing cells. This property manifests itself in their unique pentagonal capsid structure. Adeno-associated CRISPR mediated delivery systems have an impressive reputation for disease model formation, drug discovery, and existing diseases therapy (192). More importantly, the AVs of cancer treatment have become widely used in cancer therapy since they have the capacity to determine cancer cells and the virus that has obsessed as oncolytic therapy. Maddalo and his colleagues used the AdV vector and a CRISPR/Cas9 system to intentionally create Eml4-ALK gene rearrangement in a live organism. Such targeting resulted in the establishment of a mice model with lung cancer that was induced by EML4-ALK gene mutation (192). In the area of drug discovery, Voets et al. demonstrated the application of AdVs against the SMAD3 gene in human lung fibroblasts and bronchial epithelial cells (193). The depleted PCSK9 gene function in mouse livers, as demonstrated by Ding et al. study, showed there was reduced cholesterol in the plasma (194). Maggio et al. pointed out this important part which can contribute to the optimization of AVs for cancer applications that use CRISPR machinery. They tested these in different cancer and non-cancer cell lines, such as HeLa, U2OS, hMSCs, and myoblasts, to find the AAVs containing the necessary ratio of both Cas9 and gRNA. Their results demonstrated that integrated vector designs, where both Cas9 and gRNA expression occur within single vector particles, are more efficient. This finding is an extremely important outcome (195).

### 4.3 Retroviruses

When durable transmission is important and main entry in the host genome is imperative, retroviruses represent a better option. Among retroviruses, significant types include γ-retroviruses and lentiviruses. These viruses, with RNA-based genomes and ability to replicate *via* reverse transcriptase, provide something additional that is unique to them. Whereas γ-retroviruses are infrequently used owing to their inability to transduce only actively dividing cells, β-retroviruses can easily penetrate the nucleus during mitosis process where the nuclear disintegration takes place. Additionally, insertional activities of both γ-retro viruses and LVs occur randomly in the host genome and, therefore, can cause mutagenic and oncogenic effects. LVs are an engineered HIV-1 that exhibit a particular pattern of viral infection and replication, thus requiring handling in a very safe manner. A third-generation lentiviral system was established to enhance researchers’ safety. This system utilizes four plasmids for the formation and packaging of lentiviral particles: one plasmid expresses an Envelope protein, the second one carries Gag and Pol proteins, one plasmid has a Rev gene (a transactivating protein), and the last one encodes the STOP codon or a gene to be expressed (196). Annunziato et al.’s study claimed it was exciting to look into CRISPR machinery being delivered through lentiviruses. The scientists developed a new approach for generating lobular breast adenocarcinoma in female mice by subcutaneously injecting lentiviral vectors generated with Cre recombinase and/or CRISPR/Cas9 systems into their intraductal space. They employed E1a7.2 Cre driver mice with conditional allele of Cdh1, which codes E-cadherin protein. Mice with mammary gland-specific silencing of E-cadherin via Cre/LoxP mediated editing can give rise to cancer stem cells that prompt the development of intraductal carcinoma. However, the exposure of the Cas9 system is associated with an immune response that reduces the efficiency of Pten knockout to the development of tumors with no intraductal histotype (197).

Human HCC was addressed by Liu et al. with the assistance of a lentivirus-mediated CRISPR/Cas9 system that selectively targets HIF-1α with sgRNA-721. Eventually LV-H721 was injected directly into the tumor bed of the subcutaneous xenograft model SMMC-7721. Later, the amounts of HIF-1α in tumor tissues were measured by the injection of lentivirus-mediated CRISPR/Cas9 system for 3 days (198). According to the study by Kim, an expression of Cas9 and sgRNA was used by the method of lentivirus and AAV to target mutant KRAS alleles in cancer cells. By (i.t.) these carcinomas xenografts inhibited tumor growth as confirmed by the research (199). In a recent study, Zhao et al., reported finding genes that are edited using lentivirus infection. It was revealed that targeted ablation of the gene BIRC5 delayed the transition from epithelial to mesenchymal states in ovarian cancer cell. This retraction was shown by the increased level of epithelial cell markers (e.g. cytokeratin 7) and the decreased level of mesenchymal markers (e.g. Snail2, β-catenin, vimentin). Concurrently, BIRC5 overexpression especially enables EMT (200). Cancer cells have a high level of miR-21 microRNA. It leads to the initiation of a cascade of abnormal cell divisions and eventually gives rise to drug-resistant metastasis. Scientists deliberately modified the pre-miRNA sequence to achieve complete suppression of miR-21 expression. These cellular features, which included cell proliferation, motility, and invasion, were noticeably reduced in two ovarian cancer cell lines. However, miR-21 was shown to directly block epithelial EMT by regulating E-Cadherin and Vimentin, as well as Slug (201).

## 5 Clinical trials of CRISPR/Cas9

The first-in-human (*ex vivo*) study of the patients with non-small-cell lung cancer was conducted in China, where the CRISPR/Cas9 gene editing system has been applied as a tool (202). Each subject in this procedure received Cas9 and sgRNA via electroporation to target the PD-1 gene in their peripheral blood T cells, which were then reintroduced into their bodies (203). The tracked T cells were found in the peripheral blood samples from the patients only few hours after receiving infusions, which implied that the method is effective and safe and could eventually contribute to high therapeutic efficacy.

White et al. shared a phase 1 clinical study that used CRISPR/Cas9 technology for three advanced and resisting cancers patients (204). Recently, Stadtmauer et al. (205) reported the findings of a Phase 1 clinical trial (NCT03399448) using CRISPR-Cas9 technology in three patients with advanced-stage refractory cancer. In this trial, they deleted the TRAC and TRBC genes, which encode components of the endogenous T cell receptor, and the PDCD1 gene, which encodes the PD-1 protein, from the patients’ T lymphocytes to enhance anti-tumor immunity. Additionally, they introduced a transgene for NY-ESO-1 to target tumors. The modified T lymphocytes were well tolerated by the patients for up to 9 months following reinfusion (205). In another clinical trial involving CD19-positive tumor cells, CAR-T cell therapy was recommended for treating relapsed hematological malignancies. This study involved integrating CARs targeting CD20, CD22, and CD19 into the TRAC locus of T cells, enhancing their ability to recognize and target CD19-expressing cells effectively (206). In a separate clinical trial (NCT03166878), the allogeneic universal CD19-specific CAR-T cells (UCART019) from gene-edited patients with relapsed or refractory CD19+ lymphoma and leukemia were given to patients using LV as a delivery vehicle at the dosage of 5E6 CAR-T cells/kg enveloped by LV. Genes such as B2M and TCR were targeted with DNA composed of CRISPR RNA constructed through electro-poration. Furthermore, in an in-clinical trial (NCT04438083), CTX130 allogeneic CRISPR-Cas9-edited T cell preparing was tested for its efficacy in treating renal cell carcinoma and hematological malignancies. Besides depletion of immune cells, this trial specifically tackled CD70 molecules (207). Ongoing trials (NCT02546167) involving CARs that target B cell maturation antigen (BCMA or CD269) in advanced myeloma have successfully eradicated both the malignant myeloma cells and the nonmalignant plasma cells that express BCMA (208).

The CRISPR/Cas system promises to be a powerful means of making people resistant to carcinogenic viruses with its in-built ability to modulate human cells. However, some viruses (HBV, HCV, HPV, and EBV) have been implicated in the beginning of cancer in humans, and they cause hepatocellular carcinoma, cervical cancer, and Barikitt myeloma, respectively (209–211).

## 6 Challenges and future outlook

Oncogenes, tumor suppressor genes, chemotherapy-resistant genes, metabolism-related genes, and genes associated with cancer stem cells collectively contribute to the initiation and progression of cancer. The ultimate aims of cancer therapy are to inhibit growth and the further spread of malignancy through rectification of mutations and re-establishment of the function of the genes which are disrupted. The rapid advancement of CRISPR/Cas9 gene editing technology has dramatically increased cancer research. The silencing of the tumor suppressor genes is a significant factor in the emergence and outcome of the cancer. By activating when normal tumor suppressor genes are not present or they are silenced and/or altered, oncogenes start and maintain tumor growth. For instance, the implementation of CRISPR/Cas9 has revolutionized cancer research via the validation of tumor suppressor genes both *in vitro* and *in vivo* (212, 213).

Nevertheless, the existing research on the development of this technology also has a couple of substantial hurdles in the way of bringing the CRISPR/Cas9 system to target cells. The main goal is to provide the intervention with maximum security and accuracy to the tumor spots. Different vehicles of delivery include VVs, EVs, nanoparticles, and exosome-based systems. It is imperative to surmount these hurdles to bring the cancer therapeutics developed with the use of CRISPR/Cas9 on par with the current stage of development (35). Nevertheless, every methodology is in its own right limited. The use of a viral vector for the delivery can be of concern to some due to the risk of possible side effects that could be caused by the presence of viral particles. A significant challenge with viral vectors, such as adenoviruses, is the presence of pre-existing immunity to certain serotypes in humans due to natural infections or vaccinations, complicating their systemic use. One potential solution is the implementation of a heterologous prime-boosting regimen, which involves administering the same antigens using different vectors (214). Moreover, repeated administrations of viral vectors can trigger neutralizing antibody responses, which may lower the effectiveness of the therapy (215). However, this problem can be mitigated through temporary immunosuppression (216). Another strategy to decrease serological recognition involves using Ad capsid chimeras, which develop by pseudotyping the adenoviral type 5 (Ad5) vector with fibers from less-immunogenic Ads, particularly Ad serotype 45 (217). The primary mechanism of virus-based gene therapy involves the effective interaction between viral fiber proteins and specific host receptors that are associated with specific virus strains or serotypes. If the target tissue has few or no suitable receptors, the effectiveness of the infection and subsequent gene delivery is diminished. Concerns regarding the potential for therapeutic genes to be acquired by non-targeted cells or tissues are another challenge with the use of viruses as vectors. In viral gene therapy, transductional retargeting is a prevalent strategy to overcome these limitations. This strategy involves engineering the viral surface proteins to bind selectively to receptors on the desired target cells, such as cancer cells (218). Therefore, future research must focus on innovative solutions such as heterologous prime-boosting regimens, temporary immunosuppression, and transductional retargeting to advance the safety and efficacy of viral vector-based gene therapy.

Besides VVs, EVs are emerging as promising carriers for CRISPR delivery due to their high biocompatibility, low immunogenicity, and excellent tissue penetrability. Their advantageous properties make them strong candidates for future clinical applications. This potential highlights EVs as a crucial area in gene therapy research, aligning with CRISPR advancements. Researchers have developed various EVs for CRISPR delivery, including unmodified, engineered, and exosome-liposome hybrid types. Challenges still hinder the use of EVs for CRISPR delivery in clinical settings, particularly concerning stability, targeting precision, and safety. To address these issues, selecting appropriate parental cell lines and implementing strict post-production protocols are essential. Despite these obstacles, the potential for optimizing EVs to deliver CRISPR systems safely and efficiently to specific target cells is highly promising, with significant implications for the advancement of precision medicine.

Although EV-based therapies demonstrate considerable potential for healthcare, they also raise ethical concerns and risks, particularly in the areas of patient consent, safety, equitable access and data privacy. Currently, there are no established ethical guidelines for the clinical application of EVs. A comprehensive approach is necessary to address these ethical issues and mitigate potential risks. This approach should include informed consent processes, regulatory adherence, robust data privacy protections, transparent communication, and ethical oversight. In addition to these ethical concerns, the heterogeneity of EVs presents another significant challenge for clinical application. EVs include various subsets such as exosomes and microvesicles, and current separation methods (such as differential centrifugation, polymer precipitation, and ultrafiltration) often fail to isolate specific subsets. This lack of standardization affects reproducibility and makes the purification process time-consuming and labor-intensive.

The safety and toxicity of EVs remain significant challenges that have often been overlooked. Tumor-derived EVs, known for their homologous affinity effect, can target tumor tissues effectively and have been extensively utilized in preclinical antitumor therapy studies. Nevertheless, other studies have demonstrated that tumor-derived EVs can inhibit the proliferation of CD8+ T cells by lowering the levels of IL-2 or inducing CD8+ T cell apoptosis through mechanisms such as Galectin 9 and the FasL-TRAIL pathway (219, 220). Moreover, tumor-derived EVs can activate signaling pathways that promote tumor growth, angiogenesis and metastasis, and help tumors evade immune vaccine recognition, thereby resisting treatment (221). Therefore, managing the balance between the protumor and antitumor effects of EVs is a critical issue that must be addressed (222). M1-type macrophage-derived EVs, known for their pro-inflammatory effects, have been utilized in anti-tumor therapy, however they also exhibit pose safety risks. For instance, M1-type EVs can induce macrophages in the liver and other organs to adopt a pro-inflammatory phenotype, potentially leading to organ damage (223, 224). In some cases, heterogeneous EVs may pose immune risks when used for disease treatment. Generally, more research is needed to assess the toxicity and side effects of EVs before being used in clinical settings. Additionally, extending the circulation time of modified EVs may provoke immune responses and pose safety risks. A thorough assessment of their potential immunogenicity and functionality is a critical component of the clinical application process.

The successful transition of EV-based therapies from the lab to clinical settings depends on overcoming challenges related to heterogeneity, scale-up, and precise targeting. Ongoing research holds the promise of revolutionizing treatments with engineered EVs across various diseases. To fully realize their potential, a multidisciplinary approach, incorporating advanced manufacturing, rigorous clinical trials, and innovative characterization methods, will be essential. Moreover, translating EV-based genetic engineering into clinical use and commercializing these therapies require strong collaboration between industry, academia, and regulatory bodies. Such partnerships form a synergistic ecosystem that accelerates innovation and enhances patient outcomes across various healthcare fields.

## 7 Conclusion

Ultimately, this review explores the latest technological advancements in the application of CRISPR/Cas-based gene repair systems for the treatment of cancer, with a special attention given to EVs and viral vectors. We have given a comprehensive analysis of the existing literature that shows that EVs and viral vectors are efficient vehicles that carry the CRISPR/Cas components for the targeted delivery into cancer cells. Thus, we aim to fill the gap by appraising the current status of carrier systems for CRISPR/Cas in cancer therapy thereby supporting the importance of EVs and viral vectors in accomplishing specific genome editing and targeted therapy. This approach paves the way for personalized cancer treatment.

These findings have a profound impact, both intellectually and physiologically, marking the beginning of a new era in personalized cancer treatments and enhancing our understanding of cell transfer and genome editing mechanisms. Nevertheless, it is equally significant to realize that there are some flaws in this evaluation, for example, the scope of the literature and the difficulties, if not impossibility, in assessing the effectiveness and safety of the innovative distribution systems. However, more innovative techniques, refined delivery methods, and rigorous preclinical and clinical trials are essential. This review highlights the significance of the issue and underscores the need for further exploration. It aims to inspire researchers to discover new solutions that could improve outcomes and introduce novel principles in cancer treatment, thereby making a substantial contribution to the field of precision oncology.
